# The Role of Levodopa Challenge in Predicting the Outcome of Subthalamic Deep Brain Stimulation

**DOI:** 10.1002/mdc3.13825

**Published:** 2023-07-11

**Authors:** Robin Wolke, Jos Steffen Becktepe, Steffen Paschen, Ann‐Kristin Helmers, Dorothee Kübler‐Weller, Jinyoung Youn, Dana Brinker, Hagai Bergman, Andrea A. Kühn, Alfonso Fasano, Günther Deuschl

**Affiliations:** ^1^ Department of Neurology UKSH, Christian‐Albrechts University Kiel Kiel Germany; ^2^ Department of Neurosurgery UKSH, Christian‐Albrechts University Kiel Kiel Germany; ^3^ Movement Disorder and Neuromodulation Unit, Department of Neurology Charité–Universitätsmedizin Berlin Germany; ^4^ Department of Neurology, Samsung Medical Center School of medicine Sungkyunkwan University Seoul South Korea; ^5^ The Edmond andLily Safra Center for Brain Sciences (ELSC) The Hebrew University Jerusalem Israel; ^6^ Department of Medical Neurobiology (Physiology), Institute of Medical Research‐Israel Canada (IMRIC), Faculty of Medicine The Hebrew University Jerusalem Israel; ^7^ Department of Neurosurgery, Hadassah Medical Center The Hebrew University Jerusalem Israel; ^8^ Edmond J. Safra Program in Parkinson's Disease, Morton and Gloria Shulman Movement Disorders Clinic Toronto Western Hospital, UHN Toronto Ontario Canada; ^9^ Division of Neurology University of Toronto Toronto Ontario Canada; ^10^ Krembil Brain Institute Toronto Ontario Canada; ^11^ Center for Advancing Neurotechnological Innovation to Application (CRANIA) Toronto Ontario Canada

**Keywords:** deep brain stimulation, levodopa challenge, Parkinson's, subthalamic, stimulation, prediction

## Abstract

**Background:**

Deep brain stimulation of the subthalamic nucleus (STN‐DBS) is an effective and evidence‐based treatment for idiopathic Parkinson's disease (iPD). A minority of patients does not sufficiently benefit from STN‐DBS.

**Objective:**

The predictive validity of the levodopa challenge for individual patients is analyzed.

**Methods:**

Data from patients assessed with a preoperative Levodopa‐test and a follow‐up examination (mean ± standard deviation: 9.15 months ±3.39) from Kiel (n = 253), Berlin (n = 78) and Toronto (n = 98) were studied. Insufficient DBS outcome was defined as an overall UPDRS‐III reduction <33% compared to UPDRS‐III in med‐off at baseline or alternatively if the minimal clinically important improvement of 5 points was not reached. Single UPDRS‐items and sub‐scores were dichotomized. Following exploratory analysis, we trained supervised regression‐ and classification models for outcome prediction.

**Results:**

Data analysis confirmed significant correlation between the absolute UPDRS‐III reduction during Levodopa challenge and after stimulation. But individual improvement was inaccurately predicted with a large range of up to 30 UPDRS III points. Further analysis identified preoperative UPDRS‐III/med‐off‐scores and preoperative Levodopa‐improvement as most influential factors. The models for UPDRS‐III and sub‐scores improvement achieved comparably low accuracy.

**Conclusions:**

With large prediction intervals, the Levodopa challenge use for patient counseling is limited, though remains important for excluding non‐responders to Levodopa. Despite these deficiencies, the current practice of patient selection is highly successful and builds not only on the Levodopa challenge. However, more specific motor tasks and further paraclinical tools for prediction need to be developed.

Deep brain stimulation of the subthalamic nucleus (STN‐DBS) is an effective treatment for idiopathic Parkinson's disease (iPD).^1^ However, a minority of patients does not sufficiently benefit from DBS. In this study, we investigate whether the preoperative Levodopa challenge is suitable to identify DBS non‐responders prior to surgery on an individual basis and its reliability. The Levodopa challenge found its way to clinical use due to its differential diagnostic value for Parkinson's syndromes and the identification of atypical Parkinson's syndromes, which are less responsive to dopaminergic agents. Charles et al reported significant correlation between preoperative UPDRS III reduction during the Levodopa challenge and the postoperative UPDRS III reduction in med‐off stim on,^2^ later confirmed in several meta‐analyzes and studies for both, STN‐ and GPi‐DBS.^3^, ^4^, ^5^

Based on these findings, it is commonly accepted that UPDRS III reduction during Levodopa challenge may predict the STN‐DBS outcome within a short follow‐up period. This correlation of absolute data was reproduced by many groups but also relative levodopa responsiveness was found to relate to the STN‐DBS outcome. However, Zaidel et al challenged this belief regarding the relative UPDRS III reduction.^6^ Long‐term outcome was consistently found not be related with improvement during the Levodopa challenge.^7^, ^8^, ^9^, ^10^

Recent studies reported that logistic regression discriminates between DBS responders and non‐responders with up to 77% classification accuracy using a model mainly based on data of the preoperative Levodopa challenge, which led to the expectation that new statistical methods may improve individual prediction.^11^, ^12^

This study aims to reevaluate the predictive abilities of the Levodopa challenge on a large multicenter dataset of iPD patients. Data of preoperative Levodopa challenge and postoperative UDPRS III of early follow‐up examinations was analyzed systematically applying state‐of‐the‐art statistical methods.

## Methods

In this study, we combined datasets of early follow up examinations (9.15 months ±3.39 months) from University Clinic Kiel (n = 253), University Clinic Toronto (n = 98), and Charité University Clinic Berlin (n = 78). The Berlin data was collected using the MDS‐UPDRS and transformed according to standards.^13^

Insufficient DBS outcome was defined as an UPDRS III reduction of less than 33% compared to UPDRS III in med off at baseline or alternatively if the minimal clinically important improvement of 5 points was not reached.^1^, ^14^, ^15^ To examine the predictive power for single symptoms (UPDRS items) and symptom groups (sub‐scores), these were dichotomized based on clinical experience. For the tremor, the rigidity and akinesia items lateralized sub‐scores of the more affected body side were calculated. As iPD commonly shows lateralization of symptoms this promises a reduction of statistical noise. Categorical responses of a successful improvement were defined as follows: Rest‐ and action tremor were regarded as sufficiently treated if the scores of items 20 and 21 of the more affected hand in med‐off at baseline were equal to 0 (no tremor) or 1 (only slight tremor). As lateralized akinesia sub‐scores, lateralized rigidity sub‐scores, and PIGD sub‐scores consist of more than a single item that provided a logical clinical threshold for dichotomization, a reduction of less than 33% of these sub‐scores compared to pre‐operative state was considered as insufficient. Sub‐scores of the UPDRS were used according to established standards. For rest tremor of the hands the item 20 of the most affected hand and for action tremor of the hands the item 21 of the most affected hand was taken. If both hands were similarly affected the mean of both sides score was taken. The lateralized rigidity score was defined as the mean scores of the most affected body side and the head was excluded. For the lateralized akinesia score we took the mean of the items 23, 24, and 25 of the more affected body side. In case of symmetrical symptoms, the mean of the sub‐score items of both sides was considered. The postural instability and gait disorders (PIGD) score consist of the mean of the items 28, 29, 30, and 31.

Motor improvement due to Levodopa or stimulation was defined as:

**Levodopa improvement**:

preoperative scoremedoff−preoperative scoremedon

**Stimulation improvement**:

preoperative scoremedoff−postoperative scoremedoffstimon

**Relative Levodopa improvement**:

$$\frac{\text{preoperative score}\;\mathrm{med}\;\mathrm{off}-\text{preoperative score}\;\mathrm{med}\;\mathrm{on}}{\text{preoperative}\;\mathrm{med}\;\mathrm{off}}\times 100$$

**Relative stimulation improvement**:

$$\frac{\text{preoperative}\;\mathrm{med}\;\mathrm{off}-\text{postoperative}\;\mathrm{med}\;\mathrm{off}\;\text{stim}\;\mathrm{on}}{\text{preoperative}\;\mathrm{med}\;\mathrm{off}}\times 100$$

For statistical comparisons we used Pearson's Chi‐squared test for categorial comparisons and the Kruskal‐Wallis rank sum test for testing the overall differences for continuous variables of the three centers. Correlations between the Levodopa and stimulation improvement the relative Levodopa and stimulation improvement and the UPDRS III Score at baseline and the stimulation improvement were illustrated via the Pearson coefficient. For deeper dimensional analysis, we used a multi‐variate linear regression model with the stimulation improvement as dependent variable and Levodopa improvement and UDPRS‐III med off as independent variables. As traditional tests for normality increase sensitivity as the sample size increases, normality was inspected with “normal QQ‐plots” (see Fig. S1a,b). Given the absence of multi‐collinearity, beta‐coefficients of this multi‐variate linear regression model reveal change in dependent variable for every 1‐unit of change of the specific predictor variable. Common indicators for multicollinearity, such as the variation inflation factor (<3) and correlation of single variables (<0.8), might neglect slight multi‐collinearity.^16^ “Shapley”‐analysis, a game‐theoretical approach, is regarded more robust to model the relative contribution of different variables to dependent variables.^17^ Shapley‐values were calculated using the “fastshap” package for R.^18^ For predictive modeling, we applied a generalized linear regression and logistic model, XGBoost algorithms for both regression and classification, and support‐vector‐machines with polynomial kernels. The data was normalized and centered before model fitting. For hyperparameter tuning, the default grid search of the “caret” R package was used. The data was centered and scaled before model training. To adjust for class imbalances, the SMOTE algorithm was applied using 10‐fold‐10‐times‐cross‐validation to estimate the predictive power of the model on unseen data. For regression, we used the *R* ^2^ measure to evaluate our models’ performance. The sensitivity, specificity, and area under the curve (AUC) of the corresponding receiver operating curves (ROC) were reported for classification tasks. A ROC‐AUC can vary between 0 and 1, a value of greater (or less) than 0.5 is a metric for the discriminating power between two classes.

Statistical analysis and model building was carried out using the R “base” library and the “caret” and “caret ensemble” R‐package.^19^, ^20^, ^21^ For data visualization we used “ggplot2”.^22^ The code will be available upon reasonable request. This protocol was conducted following the Declaration of Helsinki and is approved by the ethics committee of the Kiel Medical Faculty.

## Results

### Clinical Data

We compared data at baseline between the centers and found significant differences in age of implantation and UPDRS III including sub‐scores between the centers (Table 1). In order to cover the largest possible range of phenotypes, the datasets were merged for further analysis and for predictive model training.

**TABLE 1:** Clinical data of the three patient groups. All scores range from 0 to 4

Characteristic	Kiel, N = 253*	Toronto, N = 98*	Berlin, N = 78*	*P*‐value
Sex				0.065**
Female	92 (36%)	23 (23%)	24 (31%)	
Male	161 (64%)	75 (77%)	54 (69%)	
Age at baseline	61 (8)	57 (7)	62 (9)	<0.001***
Rest‐tremor med off, most affected hand	1.64 (1.42)	1.37 (1.32)	1.33 (1.28)	0.13***
Action‐tremor med off, most affected hand	1.44 (1.11)	1.25 (0.80)	2.38 (0.90)	<0.001***
Rigidity score med off, most affected side	1.71 (0.79)	1.55 (0.94)	2.01 (0.75)	<0.001***
Akinesia score med off, most affected side	2.32 (0.75)	1.99 (0.77)	2.26 (0.79)	0.001***
PIGD score med off	1.80 (0.94)	1.55 (0.69)	1.64 (0.95)	0.2***

* Mean (SD).

** Pearson's Chi‐squared test.

*** Kruskal‐Wallis rank sum test.

### Data Exploration

**Figure 1:**
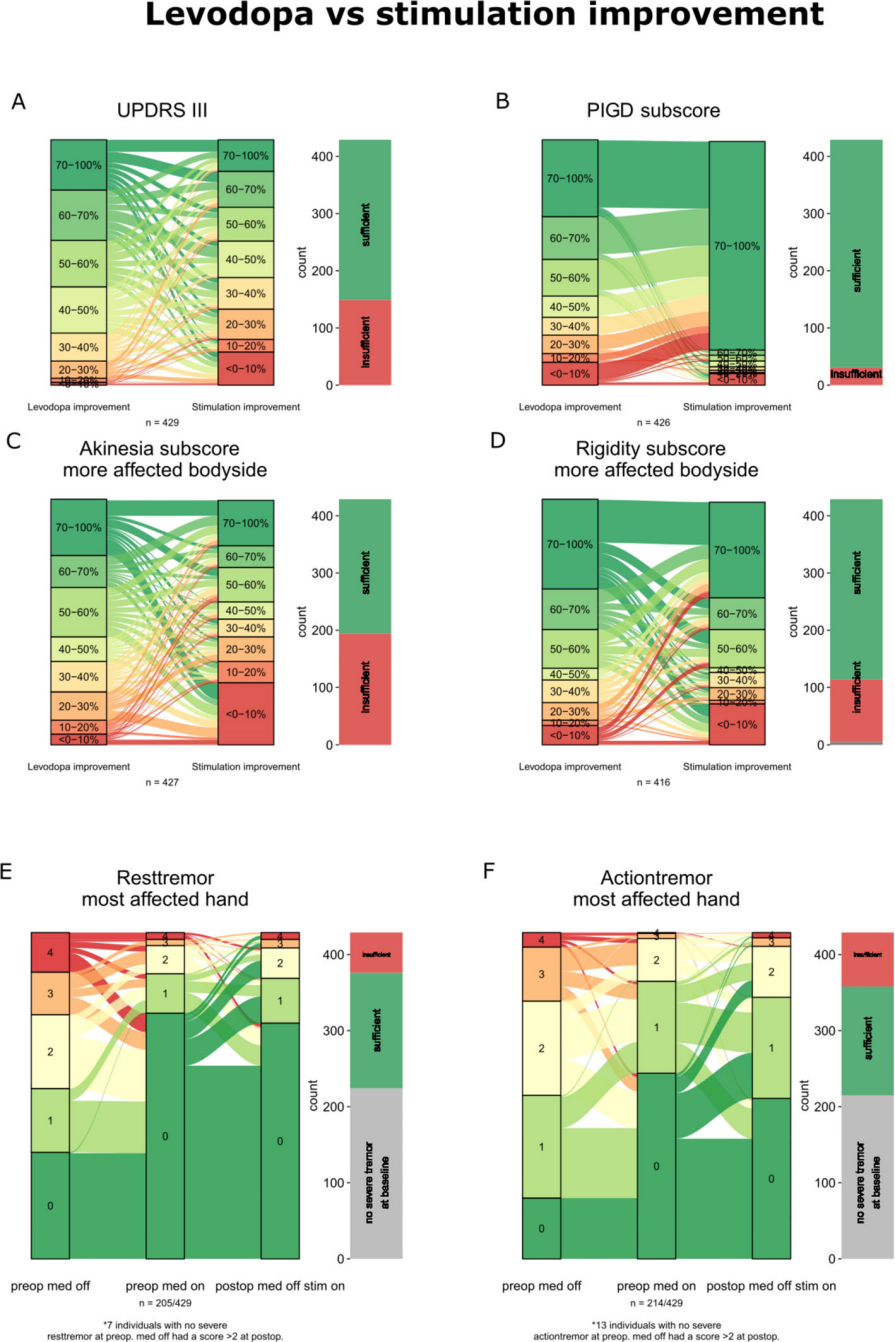
(A–D) are comparing the relative Levodopa and stimulation improvement (compare Methods section). All individuals have been divided in up to eight groups depending on the relative Levodopa and stimulation improvement. The width of the arrows between these groups indicates how many subjects switched or did not switch classes from preoperative Levodopa intake to postoperative stimulation, depending on symptom improvement. Additionally, the bars on the right indicate how many individuals reached a sufficient UPDRS III or symptom improvement (>33%, see Section 0) after DBS implantation. These Sankey diagrams emphasize the relatively poor relation between preoperative Levodopa improvement and postoperative response to stimulation. This applies specifically for the whole UPDRS III (A), akinesia (C) and rigidity (D) sub‐scores. Regarding the postural instability and gait disorders (PIGD)‐score a vast majority of patients benefit from stimulation despite the less favorable Levodopa improvement (B). Response of tremor to Levodopa and stimulation is shown for the rest (E) and action (F) tremor items of the more affected hand. The absolute scores between preoperative med off and on and postoperative med off stim on are shown (percentage changes are not meaningful here). Preoperatively, all patients included in this sub‐analysis suffered at least from a moderate tremor. Most of the cases improved to no (0) or mild (1) tremor. However, a significant portion of the good responders to Levodopa have a worse postoperative stimulation response (scores 2–4) while some of those with a poor preoperative Levodopa response have a sufficient stimulation response.

Figure 1 visualizes the relationship of relative Levodopa and stimulation improvement using Sankey diagrams. It confirms that STN‐DBS leads for the majority of patients to good therapeutic results especially treating Rigidity, Tremor and PIGD related symptoms. However, no clear relationship between relative levodopa and stimulation improvement can be inferred.

### Explanatory Analysis (Factors Explaining Stimulation Improvement)

**Figure 2:**
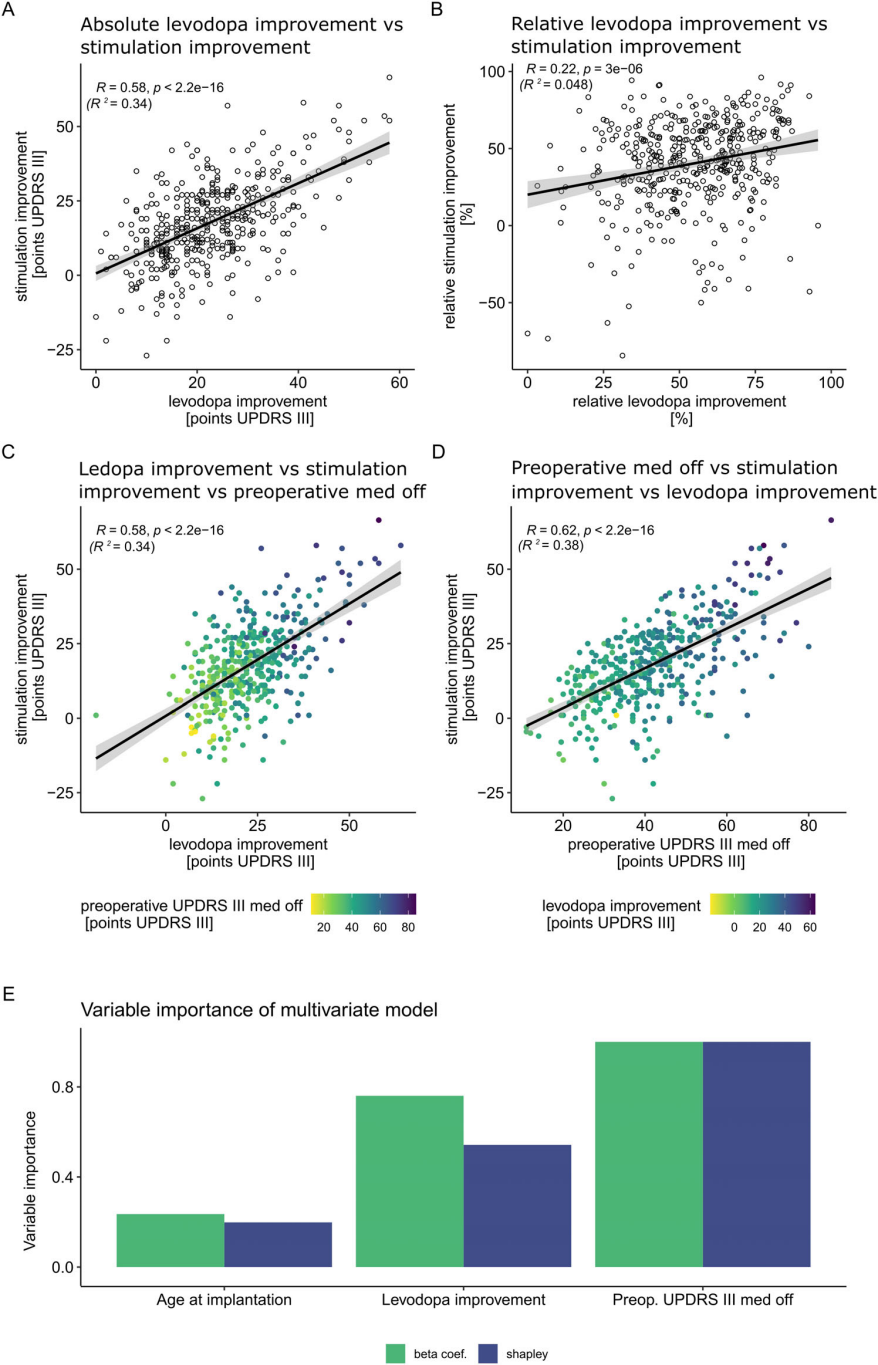
Correlation analysis between baseline parameters and the stimulation improvement. (A) shows the correlation of absolute preoperative Levodopa improvement and postoperative stimulation improvement (*r* = 0.57) and (B) the relative improvements (*r* = 0.21). In both cases there is a significant correlation (Pearson). (C) shows the correlation between the Levodopa versus stimulation improvement and, additionally, the preoperative UPDRS III med off as a color gradient. In (D) the preoperative UPDRS III med off is plotted versus the stimulation improvement and the Levodopa improvement is coded as a color gradient. (E) reveals the relatively (the values were normalized to the most important influence factor) greater importance—expressed as the linear factors or Shapley‐values—of the preoperative UPDRS III med off than the Levodopa challenge improvement.

To understand the factors linking preoperative medication and postoperative stimulation improvement, we conducted different variants of correlation analysis (Fig. 2). Firstly, the absolute Levodopa improvement was significantly related to the absolute stimulation improvement (*r* = 0.58, *P* ≤ 0.001, *R* ^2^ = 0.34, Fig 2) and both, Levodopa and stimulation improvement, were significantly correlated to preoperative UDPRS‐III in med‐off (Fig. 2 and S3). The correlation of the relative Levodopa and stimulation improvement was still significant, but much weaker than the absolute improvement (*r* = 0.21, *P* ≤ 0.001, *R* ^2^ = 0.048, Fig 2). This *R* ^2^ indicates that only 4.8% of the variance of the relative stimulation improvement is explained by the Levodopa improvement. Furthermore, preoperative UPDRS III in the med‐off and preoperative Levodopa improvement at baseline were both correlated to stimulation improvement and each other (Fig. 2C,D and S2). This relationship was analyzed in further depth by fitting a multi‐variate linear regression model, which included these two variables and the age at implantation. The expected postoperative stimulation‐result [$f\left(x\right)$] is modeled according to:

$$f\left(x\right)=0.0343-0.145\times \mathrm{age}\;\mathrm{at}\;\text{implantation}+0.31\times \text{preop}.\;\text{Levodopa improvement}+0.472\times \text{preop}.\;\text{UPDRS}\;\mathrm{III}\;\mathrm{med}\;\mathrm{off};\text{adjusted}\;R^{2}=0.43$$

The importance of the three variables can be estimated by the beta‐coefficients of the linear model. Additionally, we calculated variable importance with Shapley‐analysis known to better exclude multi‐collinearities. Both types of analyses were ranking the three factors in the same order with a slightly different magnitude (Fig. 2E). The strongest factor was the preoperative UPDRS III in the med‐off, followed by the improvement of the UPDRS III during Levodopa challenge, and lastly, the age at implantation. These three factors explain only 43% variance of the stimulation outcome, ie, roughly half of the stimulation outcome is unexplained by these variables.

### Prediction of Improvement as a Continuous Variable

Regression models [linear model (lm), Xgradient boosting tree model (xgbTree), support vector machine with polynominal kernel (svmPoly)] were used to predict the absolute stimulation improvement as a continuous variable. Dependent variables were the rest‐ and action tremor of the most affected hand, the rigidity score, the akinesia score and the PIGD‐sub‐score during preoperative med‐off and med‐on as well as the age at implantation. Measured on the average *R* ^2^ of the cross‐validation, the linear model and support‐vector‐machine performed comparably (Table S1). Therefore, we opted for the simpler and more understandable linear model (*R* ^2^ = 0.41, inter‐quartile range between 25% and 75%‐percentile [IQR^25–75^]: 0.35–0.51). Similarly, we also trained regression models to predict the relative stimulation improvement. These regression models showed a comparably low performance, with the linear model being the most successful (*R* ^2^ = 0.14, IQR^25–75^: 0.08–0.20).

### Prediction Models of Improvement as a Dichotomized Variable

Another statistical approach is to dichotomize outcomes into favorable and unfavorable outcomes. A logistic regression model defining a favorable outcome as >33% postoperative stimulation improvement showed a median ROC‐AUC of 0.66 (IQR^25–75^: 0.60–0.71) and a median specificity of 0.47 (IQR^25–75^: 0.40–0.60) during 10‐times repeated‐10‐fold‐cross‐validation. A similarly trained logistic regression model applied to predict the minimal clinically relevant improvement of 5 UPDRS III points reached a median ROC‐AUC of 0.72 (IQR^25–75^ 0.64–0.80) and a median specificity 0.50 (IQR^25–75^ 0.43–0.67).

Lastly, we also examined the classification of dichotomized rest‐ and action tremor outcome and akinesia, rigidity and PIGD sub‐score (see Table S1 and S2). Individuals with less than 1 point in rest tremor or action tremor or a rigidity‐ or akinesia score of 0 at baseline were excluded from model training. No model reached a clinically applicable ROC‐AUC and specificity. As only 26 subjects did not reach a sufficient PIGD score reduction, no model predicting the PIGD outcome could be reasonably trained despite application of Synthetic Minority Over‐sampling Technique (SMOTE).

## Discussion

Our analysis of a large multicenter dataset confirmed correlation between improvement of UPDRS III scores during preoperative Levodopa challenge and outcome after STN‐DBS. The correlation between the absolute Levodopa and absolute stimulation improvement (*R* = 0.57, *P* < 0.001) perfectly matches the first description by Charles et al^2^ (*R* = 0.58, *P* < 0.001). However, it is demonstrated that this correlation does not allow to predict an individual patient's response with clinically sufficient precision. Also, more sophisticated statistical models or artificial intelligence are unlikely to improve the prediction based on the Levodopa response. This limitation has already been suspected by Zaidel et al, but this message did not prompt further conclusions.^6^

During explanatory analysis, we found that stimulation improvement is related to both, the absolute severity of the disease in the OFF‐condition at baseline and the Levodopa improvement. An influence of the disease severity was already noted on the level of meta‐analysis and other cohorts. In contrast to previous interpretations, we found evidence that disease severity is more relevant to predict the stimulation improvement than UPDRS III reduction during Levodopa challenge.^4^, ^7^, ^8^ This is revealed by a general linear model and the estimated beta‐ or Shapley‐values (Fig. 2). The most straightforward interpretation of this notable circumstance is that—regarding the absolute values—patients with a more severe disease have greater room for both stimulation (Fig. 2) and Levodopa improvement (Fig. S2). Logically, due to this greater role of disease severity Levodopa and stimulation improvement expressed as percentage of disease severity (relative improvement) are only weakly related (Fig. 2 and S2).

The second and expected factor for the postoperative improvement is the preoperative response of clinical symptoms to Levodopa. It holds true that the more absolute UPDRS III improves after Levodopa, the better is the response to stimulation. But as outlined above the disease severity before Levodopa intake is influencing this relationship (Fig. 4).

In order to translate these findings into forecasting the result of STN‐DBS, two approaches were used. In the first, we predicted the continuous values of the UPDRS III or its sub‐scores, whereas in the second we divided the cohort into sufficient or insufficient responders to predict the individual patient's outcome. The result was not satisfying as only 43% variance of absolute stimulation improvement could be explained with this model. The individual prediction was poor as the prediction interval had a range of up to 30 points. Predicting the relative stimulation improvement was even less successful. Therefore, machine learning techniques and cross‐validation did not improve the fit of these regression models.

Subsequently, we fitted classification models to predict UPDRS III improvement due to stimulation for two dichotomized outcomes: firstly, a sufficient result to stimulation response was defined as an improvement on the UPDRS III of more than 33% and, secondly, an improvement of UPDRS III of more than 5 points. The models’ discriminating values, and sensitivity and specificity were accessed using ROC‐AUC. The mean ROC‐AUC was 0.72 for >33% improvement and 0.78 for 5‐point improvement. This result is hardly precise enough for patient counseling. A previous report used a large number of further predictors (UPDRS II, UPDRS IV, gender, age, Hoehn and Yahr stage on‐and‐off, daily Levodopa equivalent dosage, and disease duration) and used a more sophisticated pathway of separating sufficient and insufficient results. Nevertheless, they found ROC‐AUC of only 0.79, suggesting that none of those additional predictors are stronger than those used in our study.^11^, ^12^ We have compared prediction models with more or less complicated mathematical algorithms, but they did not differ significantly in their performance. There are important statistical limitations inherent to classification models. If two alternative outcomes are possible and one of them is much more frequent, the a‐priori statistical likelihood is unbalanced. We compensated for this by applying oversampling methods, but this did not sufficiently improve the result.

We conclude that statistical approaches can theoretically improve the overall outcome prediction but are unlikely to improve the insufficient prediction of individual prognosis of DBS‐results merely based on the Levodopa challenge. It seems to be a problem of the Levodopa challenge rather than a problem of statistics.

### Limitations

This study is focusing on the value of the Levodopa challenge for prediction. First of all, our analysis is based on the assumption that the Levodopa challenge itself was conducted properly. Although all centers followed a similar formal protocol of the Levodopa challenge, we could not account for possible interrater variability in the UPDRS III, which, however, is known to be within a tolerable range.^23^ Secondly, an accurate placement of the DBS leads is a prerequisite, but we did not have the data to systematically control for the lead positioning. In our analysis, we assumed DBS‐programming followed best clinical practice, but we could not control this factor in this retrospective study either. Additionally, we could not consider clinical features beyond UPDRS III (eg, psychological effects), genetics and intra‐ and perioperative complications. These may improve predictions in the future. These are all limitations of our study, but on the other hand, the contributing centers are working according to international standards to which members of the teams have contributed in different combinations over the years.^1^, ^24^, ^25^, ^26^, ^27^ The general rules for performing these tests and management of the patients are therefore highly similar. Furthermore, current data was gathered as UPDRS III. If findings of this analysis still hold true for MDS‐UPDRS needs to be studied further knowing that UPDRS III and MDS‐UPDRS III are highly correlated (*R* = 0.96).^28^ Concerning statistical methods, machine learning algorithms “xgboost” and “svm” are limited by choice of hyper‐ and tuning parameters. But even with deepened finetuning of models, the uncovering of new relations seems unlikely. Finally, even the relatively large number of 429 cases included in our analysis might still be insufficient to cover heterogeneity among patients suffering from iPD.

### Impact of these Findings

**Figure 3:**
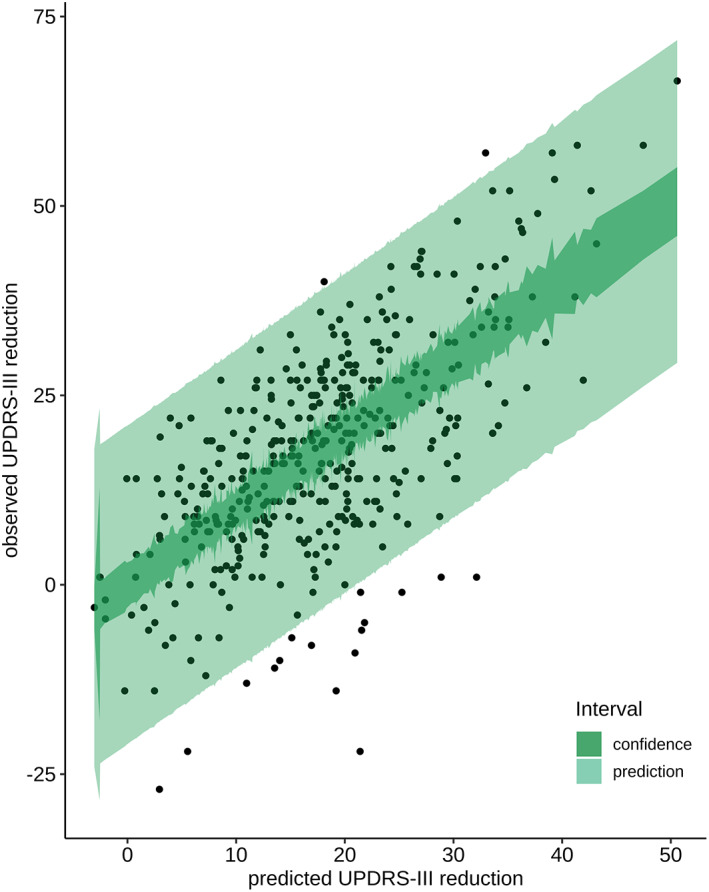
This scatter‐plot shows the observed versus predicted UPDRS III reduction after surgery of an exemplary linear regression model. In order to illustrate the concepts of confidence and prediction interval the dependent and independent variables were not scaled or normalized. The important message for prediction is that this model shows that for the same UPDRS III improvement due to medication at baseline very different postoperative stimulation responses can be obtained—even after statistical optimization. The 95%‐confidence and the 95%‐prediction interval are indicated.

The question is why the Levodopa challenge has been regarded as a particularly useful predictor of DBS outcome for more than two decades despite an earlier paper already mentioning this question.^6^ Several causes may come together here. First, a possible confusion between statistical concepts: confidence interval and prediction interval. The confidence interval indicates the uncertainty of the mean of a prediction, while the prediction interval describes the range where 95% of new individual observations will fall into. Figure 3 shows that the result of a prediction based on the best linear model is still an interval of more than 30 points on the UPDRS III scale. While deciding together with the patient for or against stimulation, our teams meanwhile avoid the strict statement that “the response to STN‐DBS will be comparable to the Levodopa response.” Secondly, we confirmed that the severity of disease is a second factor contributing to the prediction of the absolute result of STN‐DBS for short‐term follow‐up. Based on evidence of considerable limitations of the Levodopa challenge, the question arises if it should be abandoned.

**Figure 4:**
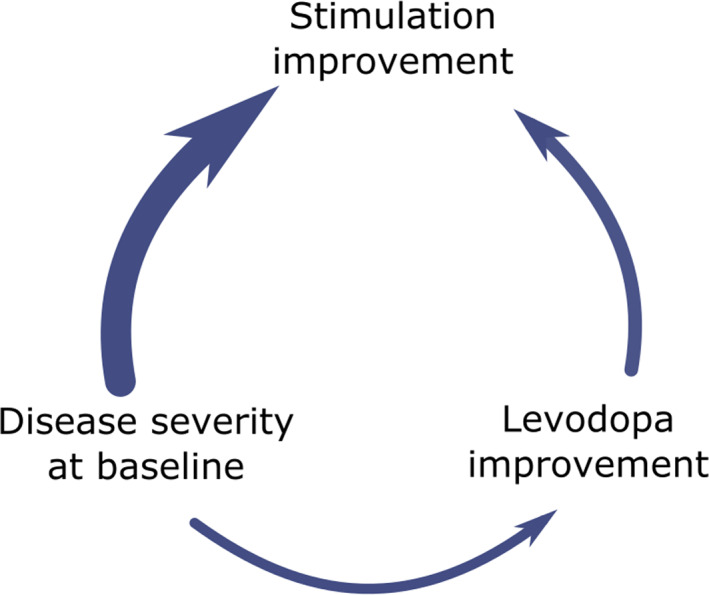
This figure recaptures the relationships of disease severity at baseline, Levodopa and stimulation improvement found during the explanatory analysis. Disease severity is significantly correlated to Levodopa improvement (Fig. S2A) and stimulation improvement (Fig. 2D). Secondly, as expected the Levodopa improvement and stimulation improvement are also related (Fig. 2A). The analysis of this 3‐dimensional relationship revealed that the disease severity is the more important factor for stimulation improvement (Fig. 2E).

Our results provide evidence that the absolute and relative Levodopa improvement inherits a low predictive capability. Therefore, it can be questioned if the clinical application of the Levodopa challenge prior to DBS is an unnecessary burden for patients and caregivers. It can be argued that general Levodopa responsiveness can be deducted from anamnesis leading to optional testing for many patients. Moreover, the Levodopa challenge is only one part during the referral process of patients toward DBS.

Besides the overall burden of Parkinson's disease, the profile of specific symptoms is decisive. For example, tremor is known to improve due to DBS independently of the Levodopa challenge result even in the long run.^29^, ^30^ This is very similar for rigidity of the extremities. Again, long‐term studies show that there is a sustained improvement for this specific symptom.^29^ Further, there is excellent improvement in motor fluctuations, a complication that cannot be accessed with the Levodopa challenge.^29^

However, there are symptoms for which an assurance of Levodopa response can be beneficial. Patients with relevant gait and balance disturbances unresponsive to Levodopa are usually excluded from surgery and only those remain who have a good response. Therefore, the excellent result of the PIGD‐score of our patients (Fig. 1) is most likely the result of an a‐priori selection.

The formal Levodopa responsiveness is a standard inclusion criterion in clinical trials on DBS in Parkinson's disease. Thresholds of Levodopa responsiveness serve as selection criteria which enhance exclusion of atypical or other causes of Parkinsonism. Furthermore, UPDRS III scores before the Levodopa challenge is after dopamine withdrawal ie, the worst “off”‐state of the patient. Our explanatory analysis provides evidence that this UPDRS score is—among those investigated—the most influential predictor determining DBS outcome. Bearing in mind limitations discussed earlier, this important variable must not be left aside during scientific trials.

We would like to emphasize that it is not reasonable to simply ignore the response to Levodopa, whether it is reported by the patient or formally assessed. The current dataset is severely under‐sampled regarding the group of Levodopa non‐responders, just as in the majority of DBS studies.^27^, ^31^, ^32^, ^33^, ^34^, ^35^, ^36^, ^37^, ^38^ There are insufficient data from patients who underwent surgery with a Levodopa response below a threshold of 33%. Therefore, whether the formal threshold of Levodopa responsiveness should be adjusted, cannot be answered. Most of the patients in this dataset with an insufficient preoperative Levodopa response—as far as the retrospective data can be interpreted—were probably operated due to medication resistant tremor or severe motor fluctuations which cannot be captured by the UPDRS III (see Fig. S4 and Table S4).

### Outlook

Although our results shed light on the limitations of prediction of the Levodopa challenge, our study cannot identify the factors causing this high variability in responses and we can only hypothesize. These factors could include the limited ability to standardize the pharmacologic challenge and the limited reliability of the UPDRS.

A more general issue could be that we have only imperfect tools to capture the relevant clinical change for specific symptoms. For example, retrospective video‐assessment of a patient's improvement in turning around while walking during the Levodopa challenge achieved better results for prediction of improvement in freezing of gait than the item 14 of the UDPRS III and the total UPDRS III.^39^ Also, other scales may be worth exploring. For example, improvement on the Berg Balance Scale correlated significantly with postoperative improvement in balance.^40^ The search for new predictors, such as imaging and DBS‐specific neurophysiology, is particularly interesting. Horn et al reported that successful DBS was associated with specific structural connectivity of the stimulated area.^41^ A retrospective analysis demonstrated significant correlation between the basal ganglia resting‐state and the clinical outcome.^42^ Additionally, local field potential recordings have predictive abilities with respect to DBS outcome. The span of beta oscillations of the DBS electrode tract is related to DBS outcome.^43^ Additionally, clustering methods have been used to localize a probabilistic sweet spots for DBS lead placement leading to improved motor symptoms.^44^ While the role of different genetic profiles in Parkinson's disease might be of importance in future therapy, current data are not yet sufficient to relate genetics and DBS response.^45^ Although the current study focused on predicting motor outcome, it can be argued that outcome prediction should be multidimensional, eg, including measures of general quality of life and non‐motor predictors.^46^, ^47^

### Conclusion

The future must be to develop a more holistic approach unifying clinical and paraclinical predictors to forecast the outcome of DBS surgery and to provide further evidence in an individualized perspective. It would be desirable if these attempts would be founded on a collaborative database that encompasses a wider variety of potential predictors. Until then the strict border of relative Levodopa improvement measured with the UPDRS (or MDS‐UPDRS) will exclude some patients from potential benefits of DBS. Nevertheless, it is currently a necessity to assure the homogeneity of study populations in interventional studies. This study also showed that clinical principles need to undergo constant reevaluation.

## Author Roles

(1) Research project: A. Conception, B. Organization, C. Execution; (2) Statistical Analysis: A. Design, B. Execution, C. Review and Critique; (3) Manuscript: A. Writing of the first draft, B. Review and Critique.

R.W.: 1A, 1B, 1C, 2A, 2B, 3A

G.D.: 1A, 2A, 2C, 3A, 3B

J.Y.: D.K., J.B.: 1B, 2C, 3B

S.P.: 2C, 3B

D.B.: 2C, 3B

A.K.H.: 2C, 3B

H.B.: 2C, 3B

A.A.K.: 2C, 3B

A.F.: 2C, 3B

## Disclosures

**Ethical Compliance Statement:** The authors confirm that this work did not require approval from an institutional review board because it was based on anonymized data. Consent from individual patients to use anonymized data for further research purposes was obtained at the time of inclusion in the databases. We acknowledge that we have read the Journal's statement on ethical publication issues and confirm that this work is in accordance with these guidelines.

**Funding Sources and Conflicts of Interest:** No funding was obtained for writing this article.

**Financial Disclosures for the Previous 12 Months**: RW, and DB: no Disclosures. JB received honoraria from Ipsen for serving as a speaker. SP received lecture fees from Medtronic and Insightec, travel grants from Desitin, travel and educational grants from AbbVie and Boston Scientific. AKH received Travel grants from Boston Scientific and speaking honoraria from Medtronic. JY declared speaker's honoraria from SK chemicals, Myung‐in Pharm, Boston Scientific and Medtronic. HB serves as a consultant for AlphaOmega, Israel. AAK received honoraria from Medtronic, Boston Scientific, Teva and serves as a consultant for Medtronic, Boston Scientific. AF received research support from Medtronic, Boston Scientific, University of Toronto, Michael J. Fox Foundation for Parkinson's Research and Dystonia Medical Research Foundation, and honoraria from Abbott, Brainlab, UCB pharma, Medtronic, Novartis, Boston Scientific, AbbVie, Ipsen, and Sunovion for serving as a speaker and/or consultant. GD has served as a consultant for Cavion and Functional Neuromodulation. He has received royalties from Thieme publishers and funding by the German Research Council (SFB 1261, T1). DKW und AAK: Funding by the Deutsche Forschungsgemeinschaft (DFG, German Research Foundation)—Project‐ID 424778381—TRR 295. AAK: Funded by the Lundbeck Foundation (Grant No. R336‐2020‐1035) and by the Deutsche Forschungsgemeinschaft (DFG, German Research Foundation) under Germany's Excellence Strategy—EXC‐2049—390688087.

## Supporting information


**Figure S1a.** Age of implantation (A), stimulation improvement (B), levodopa improvement (C), and the UPDRS III med off at baseline (D) in relation to its relative distribution (y). These are the dependent and independent variables of the multivariate model referred to in Fig. 2. The dotted red line marks the default normal‐ distribution.
**Figure S1b.** QQ‐plots of dependent and independent variables of the multivariate model referred to in Fig. 2. Normality can be assumed based on these plots.
**Figure S2.** Correlation plots of age at implantation and (A) stimulation improvement, (B) levodopa improvement and (C) the preoperative UPDRS III med off score.
**Figure S3.** Correlation plots of the absolute preoperative UPDRS III med off score and (A) absolute levodopa improvement which are highly related, (B) the relative levodopa and (C) the relative stimulation improvement which are less related.
**Figure S4.** This boxplot shows that the formal Levodopa non‐responders (n = 53) had significant higher tremor scores at baseline with medication than the formal Levodopa responders.
**TABLE S4.** *R* ^2^ of linear model 10‐times‐10‐fold‐crossvalidation
**TABLE S5.** Outcomes after dichotomization. *Differences in n arise from patients with sub‐score = 0 at preoperative UPDRS‐III med off. **for these two independent variables the severe class imbalance was regarded compromising. *** difference in n due to missing data
**TABLE S6.** Performance of classification models 10‐times‐10‐fold‐crossvalidation, *median (IQR^25–75^). **The model was included to illustrate the relationship of Levodopa improvement and postop. med on stim on improvement
**TABLE S7.** Characteristics of formal levodopa non‐responders. Values as mean (SD)

## Data Availability

The code used for statistical analysis is available on reasonable request. The data itself cannot be shared due to privacy regulations.
