# Supplementary material for: The Role of Levodopa Challenge in Predicting the Outcome of Subthalamic Deep Brain Stimulation
Source: Mov Disord Clin Pract. 2023 Jul 11;10(8):1181–91. doi: 10.1002/mdc3.13825 (PMC10450242; doi:10.1002/mdc3.13825)
Supplement: Supplementary file 1 — Figure S1a. Age of implantation (A), stimulation improvement (B), levodopa improvement (C), and the UPDRS III med off at baseline (D) in relation to its relative distribution (y). These are the dependent and independent variables of the multivariate model referred to in Fig. 2. The dotted red line marks the default normal‐ distribution. Figure S1b. QQ‐plots of dependent and independent variables of the multivariate model referred to in Fig. 2. Normality can be assumed based on these plots. Figure S2. Correlation plots of age at implantation and (A) stimulation improvement, (B) levodopa improvement and (C) the preoperative UPDRS III med off score. Figure S3. Correlation plots of the absolute preoperative UPDRS III med off score and (A) absolute levodopa improvement which are highly related, (B) the relative levodopa and (C) the relative stimulation improvement which are less related. Figure S4. This boxplot shows that the formal Levodopa non‐responders (n = 53) had significant higher tremor scores at baseline with medication than the formal Levodopa responders. TABLE S4. R 2 of linear model 10‐times‐10‐fold‐crossvalidation TABLE S5. Outcomes after dichotomization. *Differences in n arise from patients with sub‐score = 0 at preoperative UPDRS‐III med off. **for these two independent variables the severe class imbalance was regarded compromising. *** difference in n due to missing data TABLE S6. Performance of classification models 10‐times‐10‐fold‐crossvalidation, *median (IQR25–75). **The model was included to illustrate the relationship of Levodopa improvement and postop. med on stim on improvement TABLE S7. Characteristics of formal levodopa non‐responders. Values as mean (SD) [file MDC3-10-1181-s001.docx]

Supplementary Material

**The role of levodopa challenge in predicting the outcome of subthalamic deep brain stimulation.**

Robin Wolke MD^1^, Jos Becktepe MD^1^, Steffen Paschen MD^1^, Ann-Kristin Helmers MD, PhD^2^, Dorothee Kübler-Weller MD^3^, Jinyoung Youn MD^4^, Dana Brinker MD^1^, Hagai Bergman MD, PhD^6^, Andrea A. Kühn MD, PhD^3^, Alfonso Fasano MD, PhD^5^, Günther Deuschl MD, PhD^1^

***Supplementary*** ***Figure 1a:*** Age of implantation (A), stimulation improvement (B), levodopa improvement (C), and the UPDRS III med off at baseline (D) in relation to its relative distribution (y). These are the dependent and independent variables of the multivariate model referred to in Figure 2. The dotted red line marks the default normal- distribution.

***Supplementary*** ***Figure 1b:*** QQ-plots of dependent and independent variables of the multivariate model referred to in Figure 2. Normality can be assumed based on these plots.

A B C

***
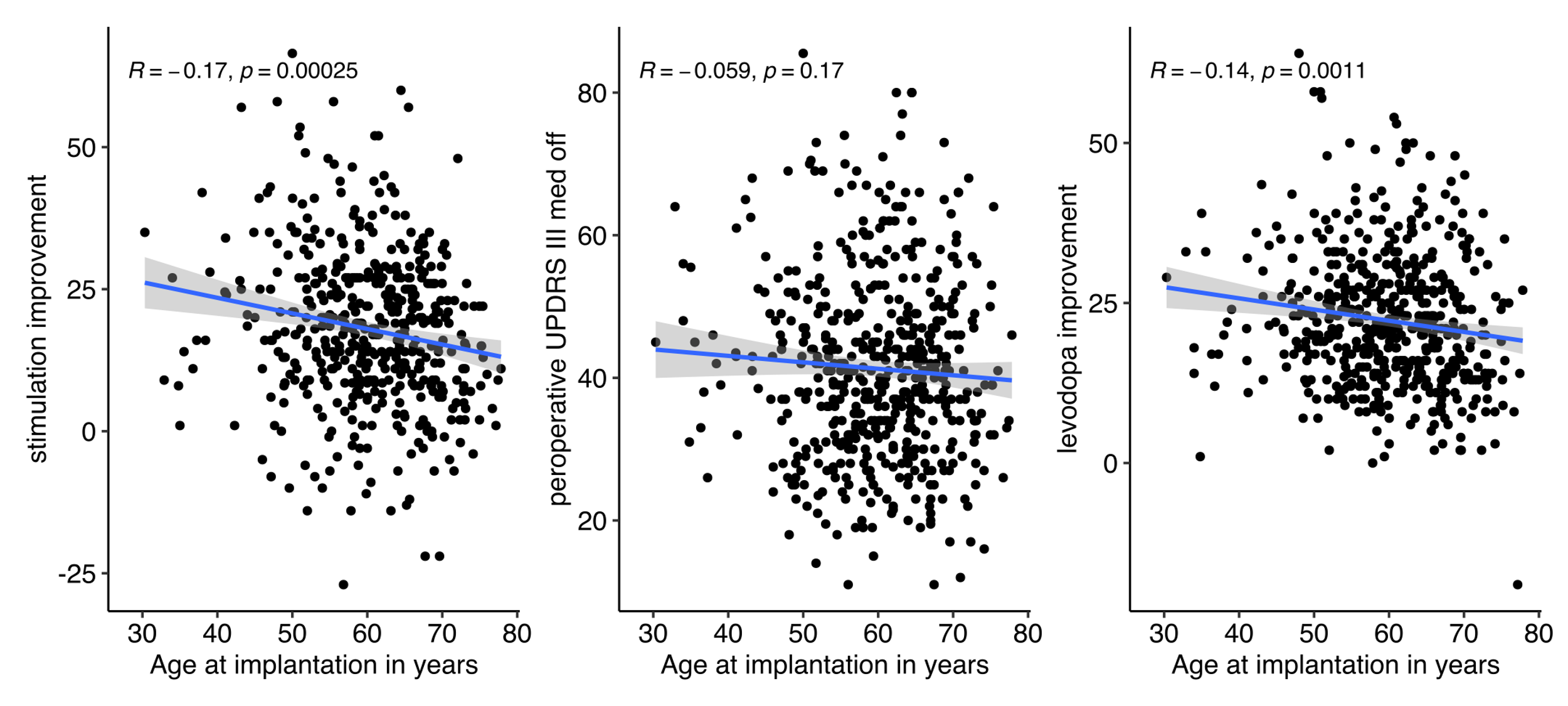
Supplementary Figure 2.*** Correlation plots of age at implantation and (A) stimulation improvement, (B) levodopa improvement and (C) the preoperative UPDRS III med off score.

A B C

***Supplementary Figure 3.*** Correlation plots of the absolute preoperative UPDRS III med off score and (A) absolute levodopa improvement which are highly related, (B) the relative levodopa and (C) the relative stimulation improvement which are less related.

|  | Min | 1st Qu. | Median | **Mean** | 3rd Qu. | Max. | N° resamples |
| --- | --- | --- | --- | --- | --- | --- | --- |
| stimulation improvement | 0.14 | 0.35 | 0.41 | **0.42** | 0.51 | 0.67 | 100 |
| relative stimulation improvement | 0.001 | 0.08 | 0.13 | **0.14** | 0.20 | 0.35 | 100 |

***Supplementary table 4.*** *R^2^ of linear model 10-times-10-fold-crossvalidation*

|  | Patients with | | Ratio insufficient/ sufficient outcome | Number of cases included* |
| --- | --- | --- | --- | --- |
|  | sufficient outcome | insufficient outcome |  |  |
| UPDRS III (>5 points reduction) | 366 | 63 | 0.17 | 429 |
| UPDRS III (>33% reduction) | 280 | 149 | 0.53 | 429 |
| Rest tremor (>1 Point) | 152 | 53 | 0.29 | 205 |
| Action tremor (>1 Point) | 152 | 62 | 0.38 | 214 |
| Rigidity | 315 | 109 | 0.26 | 424 |
| Akinesia | 235 | 194 | 0.45 | 429 |
| PIDG** | 398 | 26 | 0.07 | 424 |

***Supplementary table 5.*** *Outcomes after dichotomization. *Differences in n arise from patients with sub-score = 0 at preoperative UPDRS-III med off. **for these two independent variables the severe class imbalance was regarded compromising. *** difference in n due to missing data.*

|  | ROC* | Sensitivity* | Specificity* | Best model | N° resamples |
| --- | --- | --- | --- | --- | --- |
| UPDRS III (>33% reduction) | 0.66 (0.60-0.71) | 0.71 (0.71-0.79) | 0.47 (0.40-0.60) | logistic | 100 |
| UPDRS III (>5 points reduction) | 0.72 (0.64-0.80) | 0.76 (0.70-0.79) | 0.50 (0.43-0.67) | logistic | 100 |
|  |  |  |  |  |  |
| Rest tremor (<=1 Point) | 0.57 (0.47-0.66) | 0.75 (0.63-0.94) | 0.20 (0.00-0.40) | svm | 100 |
| Action tremor (<=1 Point) | 0.67 (0.56-0.76) | 0.68 (0.60-0.80) | 0.50 (0.34-0.67) | svm | 100 |
| Rigidity (>33% reduction) | 0.70 (0.65-0.75) | 0.75 (0.69-0.79) | 0.55 (0.45-0.64) | logistic | 100 |
| Akinesia (>33% reduction) | 0.66 (0.60-0.71) | 0.72 (0.67-0.78) | 0.45 (0.37-0.53) | logistic | 100 |

***Supplementary table 6.*** *Performance of classification models 10-times-10-fold-crossvalidation, *median (IQR^25-75^)*

***Supplementary Figure 4*.** *This boxplot shows that the formal Levodopa non-responders (n = 53) had significant higher tremor scores at baseline with medication than the formal Levodopa responders.*

|  | Clinical relevant tremor  (RT or AT>=2)  n=27 | No Tremor*  n=26 | p-value |
| --- | --- | --- | --- |
| UPDRS 3 med on at baseline | 31 (11) | 27 (8) | 0.10 |
| Actiontremor | 1.80 (0.76) | 0.65 (0.52) | <0.001 |
| Resttremor | 2.17 (1.28) | 0.19 (0.40) | <0.001 |
| Rigidity | 1.26 (0.68) | 1.02 (0.87) | 0.13 |
| PIDG | 1.02 (0.80) | 1.23 (0.87) | 0.3 |
| Akinesia | 2.14 (2.18) | 1.49 (0.66) | 0.074 |
| UPDRS 3 stim on | 23 (10) | 30 (15) | 0.084 |

*For 16 out of 26 patients, the original indication for surgery could be found in the patients reports. In 14 cases severe fluctuations with ON-OFF phases were mentioned as main indication, in one case the patient received a reimplantation of STN-electrodes and in one case poor levodopa response was attributes to the partly removal of the stomach.

***Supplementary table 7.*** *Characteristics of formal levodopa non-responders. Values as mean (SD).*
